# Supplementary material for: Uneven consequences of global climate mitigation pathways on regional water quality in the 21st century
Source: Nat Commun. 2024 Jun 27;15:5464. doi: 10.1038/s41467-024-49866-x (PMC11211422; doi:10.1038/s41467-024-49866-x)
Supplement: Supplementary file 1 — Supplementary Information [file 41467_2024_49866_MOESM1_ESM.pdf]

Supplementary Information for

Uneven consequences of global climate mitigation pathways on regional water quality in the 21st century

Minjin Lee[1*], Charles A. Stock[2], Elena Shevliakova[2], Sergey Malyshev[2], Maureen Beaudor[3], Nicolas Vuichard[4]

[1]Program in Atmospheric and Oceanic Sciences, Princeton University; Princeton, NJ 08540, USA.
[2]NOAA/Geophysical Fluid Dynamics Laboratory; Princeton, NJ 08540, USA.
[3]High Meadows Environmental Institute, Princeton University, Princeton, NJ, 08540, USA.
[4]Laboratoire des Sciences du Climat et de l'Environnement (LSCE), CEA–CNRS–UVSQ, Gif-sur-Yvette, France.

[*]Correspondence to Minjin Lee. E-mail: minjinl@princeton.edu

Note S1. Model performance analysis.
For cross-watershed evaluations, LM3-TAN results of river water discharge (m^3/s), nitrate, dissolved inorganic N (DIN), and dissolved organic N (DON) loads (ktN/yr) and concentrations (mgN/l) are compared with measurement-based estimates from 53 of the world's major rivers (Tables S1-S3)[1,2]. We report the Pearson correlation coefficient (r) between the log-transformed modeled and measurement-based estimates across the 53 rivers and the prediction error computed as the difference between the modeled and measurement-based estimates of loads expressed as a percentage of the measurement-based load (Table S4). The river results are presented for 1) the year 1990, 2) the range by using annual results for the period 1990-2000, and 3) the 1990-2000 average.

With necessary changes in the model forcings to make future projections (see Methods), we note three parameter changes from the global implementation of LM3-TAN[3]. The three parameters are 1) the reaction rate constant for freshwater nitrification[3,4] from 0.51 to 0.1 within a reported range of 0.04-3 1/day in Bowie et al. (1985) and references there[5] 2) the minimum reaction rate constant of freshwater denitrification[3,4] from 0.034 to 0.18 within a reported range of 0.034-117 1/day in Alexander et al. (2009)[6], and 3) the calibration factor for soil dissolved organic matter[3,4] introduced in Lee et al. (2014)[4] from 1 to 150.

With this minor calibration refinements, LM3-TAN is found to accurately capture observed water discharge, nitrate, DIN, and DON concentrations and loads from 53 large rivers influenced by various climate and socioeconomic conditions with skill comparable to empirical approaches (Tables S1-S4, Fig. S1). We note the degradation of fit relative to the previous global implementation of LM3-TAN[3]. The primary difference is the forcings used for the two studies. The previous study used atmospheric forcing from an atmospheric reanalysis product that integrates observations and model results to reconstruct historical forcing over a relatively short time period. The current application, however, is focused on climate change projections. This requires a global climate model capable of simulating the multidecadal evolution of the mean climate as it responds to changing greenhouse gasses, aerosols, and land use changes without any data assimilation (as there is no data to assimilate decades in the future). Global climate model projections are thus generally most skillful at continental scales and above (consistent with their intended global change applications) but can have significant regional biases[7]. This is expected to result in some degradation in fit, but has the benefit of enabling projections into the future. Such degradation resulting from simulated climate forcings is also commonly shown in other river N modeling studies (e.g., R^2 decreases from 0.60 to 0.54 when switching observed hydrology to modeled hydrology in Fig. 3 of Mayorga et al. (2010)[8]).

For global evaluations, LM3-TAN results of river DIN and DON loads and soil N storage are compared with published estimates[8-11]. Global amounts of river DIN and DON loads are found to be consistent with published ranges (Table S4). In addition, simulated global soil N storage during the 1980s-1990s are 86 PgN and 86-87 PgN without and with manure applications, which are within published ranges of 95 (70-820) PgN/yr in Post et al. (1985) and references there[10].

Although observations of global and large regional land N storage and flux changes over long time periods are not available for model validation, global terrestrial carbon (C) changes are often alternatively used to evaluate those of N given the closely coupled C and N cycles[3]. LM3-

TAN results of global terrestrial C storage and flux changes on centurial to multi-year time scales are compared with estimates from large-scale constraints and atmospheric studies[12-15]. Simulated global net land C fluxes are 1.8 PgC/yr and 2.0 PgC/yr without and with manure applications for the 1990s and 1.8 PgC/yr and 1.8 PgC/yr for 2001–2004, in consistent with estimates based on global C budget constraints (e.g., 1.1 (0.5–1.8) PgC/yr for the 1990s)[13] and inverse models (e.g., 0.3–1.7 PgC/yr for 2001–2004)[14,15], albeit at the upper end. The simulated cumulative net land C source between 1750 and 2011 is 21-23 PgC (this range is due to the climate forcing differences across the three scenarios from 2005, see Methods) without manure applications and 3 PgC with manure applications. Both cases without and with manure applications are consistent with a reported uncertainty range of 30 ± 45 PgC between 1750 and 2011[12]. The simulated land-use change contribution to elevated CO_2 between 1750 and 2011 is 188-189 PgC (this range is due to the climate forcing differences across the three scenarios from 2005, see Methods) without manure applications and 176-177 PgC with manure applications. Both cases without and with manure applications are within a published range of 180 ± 80 PgC between 1750 and 2011[12].

Lastly, we note that several previous studies have demonstrated the capacity of LM3-TAN to reasonably represent temporal variability within the Susquehanna[4,16], Mississippi[17], and Korean Peninsula[18] River Basins, which are influenced by different climate, land use, and fertilizer applications. The validated results have shown simulated vegetation and litter/soil N storage changes to significantly alter seasonal to multi-year river N extremes[16,17].

Note S2. Manure application estimates in LM3-TAN.

For the 1861-2001 simulation for which manure applications were not available, LM3-TAN was used to dynamically simulate manure applications as:

$$M_{graz\_to\_past,amm} = LM3\text{-}TAN_{graz} * F_{graz} * F_{graz,amm} \quad \text{Eq. (1)}$$
$$M_{graz\_to\_past,org} = LM3\text{-}TAN_{graz} * F_{graz} * (1-F_{graz,amm}) * F_{org} \quad \text{Eq. (2)}$$
$$M_{graz\_to\_past,nit} = LM3\text{-}TAN_{graz} * F_{graz} * (1-F_{graz,amm}) * (1-F_{org}) \quad \text{Eq. (3)}$$
$$M_{crop\_to\_past,amm} = LM3\text{-}TAN_{crop} * F_{crop} * F_{crop\_to\_past} * F_{crop\_to\_past,amm} \quad \text{Eq. (4)}$$
$$M_{crop\_to\_past,org} = LM3\text{-}TAN_{crop} * F_{crop} * F_{crop\_to\_past} * (1- F_{crop\_to\_past,amm}) * F_{org} \quad \text{Eq. (5)}$$
$$M_{crop\_to\_past,nit} = LM3\text{-}TAN_{crop} * F_{crop} * F_{crop\_to\_past} * (1- F_{crop\_to\_past,amm}) * (1-F_{org}) \quad \text{Eq. (6)}$$
$$M_{crop\_to\_crop,amm} = LM3\text{-}TAN_{crop} * F_{crop} * (1-F_{crop\_to\_past}) * F_{crop\_to\_crop,amm} \quad \text{Eq. (7)}$$
$$M_{crop\_to\_crop,org} = LM3\text{-}TAN_{crop} * F_{crop} * (1-F_{crop\_to\_past}) * (1- F_{crop\_to\_crop,amm}) * F_{org} \quad \text{Eq. (8)}$$
$$M_{crop\_to\_crop,nit} = LM3\text{-}TAN_{crop} * F_{crop} * (1-F_{crop\_to\_past}) * (1- F_{crop\_to\_crop,amm}) * (1-F_{org}) \quad \text{Eq. (9)}$$

where $M_{graz\_to\_past,amm}$, $M_{graz\_to\_past,nit}$, and $M_{graz\_to\_past,org}$ is grazing-induced ammonium, nitrate, and organic N manure from grasslands applied to pasture, $M_{crop\_to\_past,amm}$, $M_{crop\_to\_past,nit}$, and $M_{crop\_to\_past,org}$ is ammonium, nitrate, and organic N manure from managed lands applied to pasture, $M_{crop\_to\_crop,amm}$, $M_{crop\_to\_crop,nit}$, and $M_{crop\_to\_crop,org}$ is ammonium, nitrate, and organic N manure from managed lands applied to cropland, $LM3\text{-}TAN_{graz}$ and $LM3\text{-}TAN_{crop}$ is dynamically simulated grazing and crop harvest, F_{graz} (0.68) is fraction of grazing applied to pasture as manure, F_{crop} (0.35) is fraction of crop harvest applied to pasture and cropland as manure, $F_{crop\_to\_past}$ (0.45) is fraction of manure from managed lands applied to pasture, $F_{graz,amm}$ (0.58) is ammonium fraction of grazing-induced manure applied to pasture, $F_{crop\_to\_past,amm}$ (0.27) is ammonium fraction of manure from managed lands applied to pasture, $F_{crop\_to\_crop,amm}$ (0.29) is ammonium fraction of manure from managed lands applied to cropland, and F_{org} (0.8) is organic N fraction of non-ammonium manure. The reported manure applications[19-21] were available for $M_{graz\_to\_past}$, $M_{crop\_to\_past}$, and $M_{crop\_to\_crop}$ for N species of ammonium and non-ammonium. All of the fractions were approximated based on the reported manure application averages for the historical period 2002-2014, except F_{org}. The simulated manure applications for 1861-2001 are shown in Fig. S11.

River	Basin Area, km^2	Discharge, m^3/s		Nitrate Conc, mg/l			Nitrate Load, kt/yr		
		Observed	Simulated	Observed	Simulated		Observed	Simulated	
Amazon	6112000	208968	84214	0.14	0.09	0.09	922.60	245.80	246.80
Amur	1855000	10908	20033	0.02	0.19	0.19	6.88	118.30	127.70
Balsas	112000	444	1884	0.19	0.13	0.15	2.66	7.52	9.05
Brazos/Colorado	221000	239	183	0.42	0.84	1.25	3.16	4.86	7.15
Chang Jiang	1808000	29427	36270	0.32	0.16	0.18	296.03	182.70	202.50
Chao Phrya	111400	882	2046	0.14	0.12	0.13	3.89	7.99	8.61
Churchill	298000	819	1863	0.01	0.02	0.02	0.26	1.26	1.25
Columbia	669000	7484	11095	0.20	0.57	0.60	47.20	199.80	209.70
Danube	817000	6564	9305	1.80	1.44	1.71	372.60	423.20	510.40
Dnepr	504000	1693	3540	0.21	1.51	1.66	11.21	169.00	193.00
Elbe	146000	752	2662	3.60	3.86	4.07	85.32	323.70	342.90
Fraser	220000	3551	8281	0.10	0.06	0.06	11.09	16.03	15.89
Godavari/Krishna	572000	4281	6941	0.17	0.27	0.31	22.95	58.80	66.60
Huang He	752000	1300	13639	2.20	0.83	0.95	90.20	355.00	414.10
Indigirka	362000	1934	2236	0.02	0.01	0.02	1.46	1.05	1.13
Indus	916000	1807	5703	2.00	0.57	0.59	114.00	103.40	112.90
Khatanga	364000	2705	3308	0.03	0.01	0.01	2.56	1.08	1.08
Kolyma	660000	4186	3265	0.04	0.01	0.01	5.28	1.02	1.11
Lena	2490000	16648	15679	0.03	0.04	0.04	15.75	21.67	22.15
Liao	219000	514	759	0.11	3.74	4.01	1.70	89.57	103.00
Loire	112000	824	2620	1.70	0.37	0.62	44.20	30.55	51.09
Mackenzie	1787000	9767	12770	0.05	0.01	0.01	16.32	2.58	2.54
Magdalena	240900	6802	6456	0.22	0.15	0.16	47.19	29.80	32.01
Mississippi	2980000	16774	20534	1.40	0.56	0.74	740.60	360.70	487.60
Mobile	113000	1903	492	0.26	0.57	0.70	15.60	8.83	10.80
Murray	1060000	251	1470	0.11	0.43	0.47	0.87	20.08	21.43
N. Dvina	348000	3488	4026	0.02	0.09	0.09	2.20	11.54	11.13
Nelson	1132000	2832	4813	0.01	0.01	0.01	1.07	1.25	1.39
Neva	282000	2549	2807	0.23	0.10	0.10	18.49	9.18	8.89
Ob	2990000	12811	18696	0.06	1.25	1.21	24.24	736.40	727.10
Odra	112000	526	993	2.42	3.42	3.60	40.17	107.10	113.90
Olenek	219000	1135	1451	0.03	0.03	0.03	1.07	1.26	1.37
Orange	1000000	360	4371	0.72	0.93	1.05	8.18	128.40	144.50
Orinoco	1100000	35991	8277	0.08	0.12	0.13	90.80	31.50	32.76
Parana	2783000	18011	36058	0.17	0.15	0.16	93.72	173.50	179.50
Pechora	248000	3488	3958	0.07	0.02	0.02	7.70	2.27	2.34
Rhine	224000	3203	5120	3.90	2.22	2.40	393.90	358.10	385.70
Rufiji	178000	1116	1599	1.30	0.29	0.29	45.76	14.43	14.69
Saint Lawrence	1020000	10686	16223	0.16	0.13	0.14	53.92	68.40	71.70
Tocantins	757000	11796	23321	0.02	0.11	0.12	5.58	84.14	86.11
Volta	394000	1167	18829	0.15	0.04	0.05	5.52	26.69	28.07
Wisla	198000	1031	2649	1.83	3.94	4.10	59.48	329.00	347.30
Yana	230800	1088	1328	0.01	0.02	0.02	0.34	0.68	0.69
Yenisey	2590000	19660	17973	0.02	0.13	0.13	12.40	71.10	72.40
Yukon	849000	6342	13768	0.10	0.01	0.01	20.00	4.00	3.91
Zaire	3698000	40684	78933	0.08	0.13	0.13	102.64	318.80	321.90
Zambezi	1330000	3361	49031	0.13	0.13	0.15	13.78	206.00	232.30
Zhujiang	437000	11511	2901	0.62	0.33	0.35	225.06	30.27	32.33
Uruguay	240000	4598	125	0.23	0.14	0.20	32.77	0.57	0.76

Table S1. Measurement-based and simulated water discharge and nitrate estimates across 49 globally distributed rivers. All rivers of which basin areas are larger than 100,000 km^2 (about 10 grid cells in our 1 degree resolution) and log10 of actual discharges in m^3/s are not less than 2.2 were chosen from Meybeck and Ragu (2012)[2]. In total, five rivers were excluded due to their low discharges (23 m^3/s, Rio Grande (US) and 3 m^3 s^{-1} Colorado) and lack of representation

within the LM3-TAN river network (Brants, Don, and Hayes). When more than one data were given for a river, the data selected for the 1st line was used, since it was considered as "the most reliable and generally were obtained first hand by local engineers or scientists"[22]. When data in the 1st line was outdated (1970s-early 1980s) or reported as zero, the latest data was used if available in the next lines. When more than one data were given for a river with different basin areas, the data monitored at the location with the largest basin area (i.e., nearest to the river mouth) was used.

River	Basin Area, km^2	Discharge, m^3/s		DIN Conc, mg/l			DIN Load, kt/yr		
		Observed	Simulated	Observed	Simulated		Observed	Simulated	
Amazon	6112000	208968	84214	0.16	0.23	0.23	1054.40	606.70	609.30
Amur	1855000	10908	20033	0.45	0.42	0.42	154.80	267.20	281.00
Balsas	112000	444	1884	0.59	0.27	0.31	8.19	16.29	18.07
Chang Jiang	1808000	29427	36270	0.64	0.33	0.35	592.06	380.90	404.30
Chao Phrya	111400	882	2046	0.24	0.34	0.36	6.67	22.04	22.92
Churchill	298000	819	1863	0.11	0.03	0.03	2.84	1.79	1.75
Columbia	669000	7484	11095	0.21	0.62	0.65	49.56	217.60	227.20
Elbe	146000	752	2662	4.90	4.01	4.22	116.13	336.80	356.00
Huang He	752000	1300	13639	2.21	0.98	1.11	90.61	421.40	482.10
Indigirka	362000	1934	2236	0.03	0.07	0.07	1.71	5.10	5.30
Indus	916000	1807	5703	2.20	0.60	0.62	125.40	108.10	118.00
Khatanga	364000	2705	3308	0.07	0.06	0.06	5.97	6.45	6.47
Kolyma	660000	4186	3265	0.09	0.06	0.06	11.88	5.68	5.97
Lena	2490000	16648	15679	0.11	0.16	0.16	57.75	80.70	82.00
Liao	219000	514	759	0.28	4.35	4.58	4.54	104.10	117.70
Mississippi	2980000	16774	20534	1.44	0.86	1.04	761.76	559.10	687.00
Murray	1060000	251	1470	0.15	1.07	1.13	1.15	49.50	51.10
N. Dvina	348000	3488	4026	0.14	0.17	0.17	15.40	21.40	21.31
Neva	282000	2549	2807	0.26	0.11	0.11	20.90	10.10	9.79
Ob	2990000	12811	18696	0.66	1.30	1.26	266.64	766.30	757.50
Odra	112000	526	993	2.63	3.55	3.73	43.66	111.20	118.10
Olenek	219000	1135	1451	0.08	0.11	0.12	2.86	5.26	5.53
Orinoco	1100000	35991	8277	0.12	0.24	0.25	130.53	62.30	64.70
Parana	2783000	18011	36058	0.22	0.41	0.42	122.12	461.90	472.60
Pechora	324000	4154	3958	0.22	0.08	0.08	28.82	10.12	10.34
Rhine	224000	3203	5120	4.80	2.33	2.51	484.80	375.50	403.40
Uruguay	240000	4598	125	0.28	0.26	0.33	40.02	1.04	1.25
Rufiji	178000	1116	1599	1.40	1.04	1.06	49.10	52.62	53.40
Saint Lawrence	1020000	10686	16223	0.24	0.15	0.15	80.88	75.10	78.40
Wisla	198000	1031	2649	2.27	4.02	4.19	73.61	335.90	354.20
Yana	230800	1088	1328	0.18	0.09	0.09	6.17	3.93	3.98
Yenisey	2590000	19660	17973	0.30	0.19	0.19	186.00	106.10	107.60
Yukon	849000	6342	13768	0.13	0.06	0.06	26.00	25.85	25.35
Zaire	3698000	38052	78933	0.10	0.36	0.36	116.40	897.00	905.00
Zambezi	1330000	3361	49031	0.17	0.37	0.40	18.02	577.10	613.70
Zhujiang	437000	11511	2901	0.63	0.79	0.81	228.69	72.00	74.50

Table S2. Measurement-based and simulated discharge and DIN estimates across 36 globally distributed rivers. All rivers of which basin areas are larger than 100,000 km^2 (about 10 grid cells in our 1 degree resolution) and log10 of actual discharges in m^3/s are not less than 2.2 were chosen from Meybeck and Ragu (2012) [2]. In total, three rivers were excluded due to their low discharges (23 $m^3\ s^{-1}$, Rio Grande (US)) and lack of representation within the LM3-TAN river network (Brants and Don). When more than one data were given for a river, the data selected for the 1st line was used, since it was considered as "the most reliable and generally were obtained first hand by local engineers or scientists" [21]. When data in the 1st line was outdated (1970s-early 1980s) or reported as zero, the latest data was used if available in the next lines. When more than one data were given for a river with different basin areas, the data monitored at the location with the largest basin area (i.e., nearest to the river mouth) was used.

River	Basin Area, km^2	Discharge, m^3/s		DON Conc, mg/l			DON Load, kt/yr		
		Observed	Simulated	Observed	Simulated		Observed	Simulated	
Amazon	6112000	209315	84214	0.16	0.07	0.07	1056.15	186.20	186.90
Zaire	3698000	37524	78933	0.18	0.21	0.21	213.00	518.60	527.50
Mississippi	2926507	15776	20534	0.82	0.43	0.44	407.96	279.30	294.00
Parana	2783000	17650	36058	0.08	0.17	0.17	44.53	193.10	196.40
Lena	2490000	16581	15679	0.46	0.21	0.20	240.53	102.60	100.90
MacKenzie	1787000	9633	12770	0.10	0.10	0.10	30.38	41.02	41.01
Niger	1200000	4947	29730	0.13	0.12	0.12	20.28	115.40	116.40
Orinoco	1100000	35927	8277	0.16	0.05	0.05	181.28	13.35	13.80
Ganges	1050000	15649	17950	0.06	0.14	0.14	29.61	76.60	79.30
Saint Lawrence	1020000	10674	16223	0.03	0.03	0.03	10.10	16.45	16.57
Orange	1000000	317	4371	0.15	0.74	0.76	1.50	102.50	104.30
Yukon	831387	9491	13768	0.57	0.23	0.23	170.61	101.50	101.80
Danube	817000	6477	9305	0.60	0.41	0.43	122.56	119.50	127.80
Kolyma	660000	4186	3265	0.35	0.21	0.20	46.20	21.38	21.47
Colorado (CA)	638951	203	3733	0.56	0.17	0.17	3.59	20.22	20.46
Rio Grande (TX)	456701	145	3731	0.57	0.28	0.28	2.61	33.39	34.13
Khatanga	364000	2655	3308	0.41	0.37	0.37	34.33	38.16	37.69
Indigirka	362000	1951	2236	0.35	0.20	0.20	21.53	14.21	14.26
Yana	238000	1057	1328	0.33	0.35	0.34	11.00	14.52	14.56
Brazos, Colorado (TX)	225355	365	183	0.69	1.35	1.42	7.94	7.78	8.09
Olenek	219000	1111	1451	0.41	0.35	0.34	14.36	15.98	15.78

Table S3. Measurement-based and simulated water discharge and DON estimates across 21 globally distributed rivers. All rivers of which basin areas are larger than 100,000 km^2 (about 10 grid cells in our 1 degree resolution) were chosen from Harrison et al (2005)[1]. One river (Nile) without runoff information was excluded.

Results for the year 1990 in the 1st line<br>Ranges using annual results for the period 1990-2000 in the 2nd line<br>1990-2000 average results in the 3rd line										
		Nitrate			DIN			DON		
		Discharge	Without manure	With manure	Discharge	Without manure	With manure	Discharge	Without manure	With manure
Global river N loads, ktN/yr	LM3-TAN		12<br>10,13<br>11	13<br>11,14<br>13		18<br>16,19<br>18	19<br>17,20<br>19		14<br>13,15<br>14	15<br>13,15<br>14
	Pub.					14.5[9],18.9[8,11]			10[1]	
Model predictive capacity of spatial variation, r	Load	.72<br>.64,.74<br>.71	.68<br>.63,.70<br>.68	.68<br>.63,.70<br>.68	.74<br>.66, .77<br>.74	.71<br>.67,.73<br>.71	.71<br>.67,.73<br>.71	.78<br>.68,.78<br>.76	.54<br>.32,.56<br>.52	.54<br>.32,.56<br>.52
	Conc.		.74<br>.71,.76<br>.75	.75<br>.72,.77<br>.75		.77<br>.69,.78<br>.77	.77<br>.70,.78<br>.77		.67<br>.64,.70<br>.68	.67<br>.63,.70<br>.68
Prediction errors of water discharge and N loads	Min	-97<br>-100,-96<br>-98	-98<br>-100,-98<br>-99	-98<br>-100,-98<br>-99	-97<br>-100,-96<br>-98	-97<br>-100,-96<br>-98	-97<br>-100,-96<br>-98	-77<br>-84,-65<br>-75	-93<br>-97,-72<br>-89	-92<br>-97,-71<br>-75
	25th	15<br>-6,15<br>-1	-45<br>-56,-38<br>-46	-33<br>-47,-22<br>-32	13<br>-16,13<br>-3	-37<br>-39,-25<br>-28	-37<br>-37,-20<br>-26	10<br>-21,10<br>-2	-36<br>-52,-16<br>-31	-35<br>-52,-15<br>-31
	Med	48<br>35,56<br>52	45<br>37,125<br>60	43<br>42,143<br>66	47<br>31,50<br>37	24<br>2,30<br>16	24<br>6,35<br>26	31<br>16,39<br>23	11<br>-17,37<br>19	10<br>-15,45<br>18
	75th	129<br>108,140<br>112	385<br>321,501<br>342	389<br>369,568<br>422	122<br>102,128<br>98	214<br>151,241<br>237	227<br>163,258<br>249	106<br>78,167<br>98	202<br>155,290<br>224	211<br>162,233<br>233
	Max	1514<br>1265,<br>3234<br>1959	5166<br>3723,<br>8030<br>4354	5955<br>4280,<br>8675<br>4821	1359<br>1048,<br>1524<br>1217	4192<br>2729,<br>10053<br>5968	4330<br>2835,<br>10521<br>6343	2473<br>2017,<br>4509<br>2863	6735<br>3581,<br>15325<br>9014	6856<br>3595,<br>15672<br>9174
	IQR	114<br>100,140<br>113	430<br>371,545<br>389	421<br>404,610<br>453	110<br>100,139<br>100	251<br>179,277<br>265	264<br>189,287<br>275	96<br>78,159<br>100	238<br>194,327<br>255	247<br>202,344<br>264

Table S4. Model performance analysis of river water discharge and N. The cross-watershed analysis is based on the Pearson correlation coefficients (r) of the log-transformed modeled and measurement-based estimates across the 53 rivers[1-2] (Tables S1-S3, Note S1) and prediction error. Global amounts are also compared with published values[1,8,9,11] (Note S1).

	2000-2020 aveg					2079-2099 aveg [no CO_2 Fert.][A]				
	No manure			Manure		No manure			Manure	
	S1-2.6	S2-4.5	S5-8.5	S2-4.5	S5-8.5	S1-2.6	S2-4.5	S5-8.5	S2-4.5	S5-8.5
Atmos. dep.	63	64	64	64	64	47	57	70	57	70
Fertilizer	103	104	103	104	103	168	208	107	208	107
Manure	-	-	-	63	64	-	-	-	94	83
BNF	135	134	132	115	112	151 [125]	182 [114]	244 [106]	156 [93]	215 [87]
Harvest[B]	136	135	135	141	141	181 [164]	226 [179]	234 [156]	236 [186]	245 [163]
Fire	14	14	14	15	15	15 [13]	18 [14]	17 [13]	18 [15]	18 [13]
Denitrification[C]	81	80	81	102	104	97 [106]	107 [135]	70 [105]	149 [188]	103 [155]
Soil denitrification	42	41	42	56	58	52 [58]	54 [72]	32 [57]	84 [108]	56 [90]
DN flux to rivers	70	71	71	81	81	81 [84]	92 [103]	72 [82]	109 [127]	85 [104]
DN flux to the ocean	32	32	32	35	35	36 [36]	38 [40]	34 [34]	43 [46]	37 [39]
N flux to land storage[D]	39	40	36	52	49	38 [21]	59 [11]	66 [-25]	69 [17]	72 [-23]
Soil/litter storage[E]	86308	86302	86295	87011	87005	89152 [88064]	89679 [88021]	88961 [86660]	91199 [89491]	90457 [88111]

[A]The values in simulations without CO_2 fertilization (see Methods) are given in parenthesis.
[B]The sum of N in crop, grass, and wood harvest.
[C]The sum of denitrification rates in soils, rivers, and lakes.
[D]The land (terrestrial and freshwater) storage includes 5 vegetation storage (leaves, fine roots, sapwood, heartwood, and labile storage), 4 organic soil storage (fast and slow litter, slow and passive soil), 2 inorganic soil storage (ammonium and nitrate), and 6 freshwater storage (river DON, ammonium, and nitrate, lake DON, ammonium, and nitrate).
[E]The sum of N in two inorganic (nitrate and ammonium) soil and four organic (slow/passive soil and fast/slow litter) soil pools.

Table S5. Global terrestrial and freshwater N storage (TgN) and fluxes (TgN/yr). The physiological process-based BNF modeling approach of LM3-TAN suggests down-regulation of BNF when additional N via manure applications is available for plant uptake, in consistent with current literature (e.g., Barron, 2007[23]). While the recycled inputs (i.e., manure applications) reduce BNF and thus reduce total new N inputs into the land systems (Fig. 2), they increase total N inputs to soils which lead to modest increases in harvest.

		Food & Feed		Bioenergy		Total	
Model	Scenario	2010	2100	2010	2100	2010	2100
AIM/CGE	SSP1-26	3516	4842	0	2891	3516	7733
GCAM4	SSP1-26	3481	3297	0	6759	3481	10056
IMAGE	SSP1-26	3813<br>3274[A]	5509<br>4646[A]	56<br>34[A]	7748<br>5347[A]	3869<br>3308[A]	13257<br>9993[A]
MESSAGE-GLOBIOM	SSP1-26	3033	3044	0	9397	3033	12441
REMIND-MAGPIE	SSP1-26	3762	3862	9	8873	3771	12736
AIM/CGE	SSP2-45	3524	6490	0	7757	3524	14247
GCAM4	SSP2-45	3481	5316	0	6128	3481	11445
IMAGE	SSP2-45	3814	6543	57	2975	3871	9518
MESSAGE-GLOBIOM	SSP2-45	3033	4979	0	6815	3033	11794
REMIND-MAGPIE	SSP2-45	3770	6426	40	697	3810	7124
AIM/CGE	SSP5-Baseline	3524	7170	0	4134	3524	11304
GCAM4	SSP5-Baseline	3481	4624	0	1147	3481	5771
IMAGE	SSP5-Baseline	3814	7224	57	1857	3871	9081
REMIND-MAGPIE	SSP5-Baseline	3758	6198	11	189	3770	6387

[A]This crop production is from Popp et al. (2017)[24]. Note the difference in the crop production estimates by IMAGE between Popp et al. (2017)[24] and the SSP Database[25].

Table S6. Reported crop production (t DM/yr). Production of food/feed crops and of 2nd generation biofuel crops for the years 2010 and 2100 from the IAM marker and non-marker scenarios[25-30].

File name/ Data description (Size)	Availability
hydrography.1deg_cm2.20131203.tile1.nc, geohydrology_table.20090108.nc/ Geohydrology information for the river component of the model	https://zenodo.org/records/10962725
cover_frac_m45.20080723.nc/ Land cover information	
ground_frac_m45.20080723.nc/ Soil type information	
navy_topography.data.nc/ Topographic data	
1deg_mosaic_file_from_zhi_20nov2013.cpio/ Description of the model grid at 1 by 1 degree resolution (34MB)	
sst_data.cpio/ Sea surface temperature (50MB)	
geohydrology.20090108.nc/ Geohydrology information for the river component of the model (64MB)	
surface_reflectance.20090108.nc/ Soil surface reflectance (214 MB)	
Climate forcing (See Methods)	This forcing dataset totaling 899 GB is available from the corresponding author upon request.
Atmospheric CO2 concentration (See Methods)	https://esgf-node.llnl.gov/search/input4mips/, last access 3/2/2024
Land use and fertilizer applications (See Methods)	https://luh.umd.edu/data.shtml, last access 3/2/2024
Manure applications (See Methods)	https://zenodo.org/records/10100435
Atmospheric deposition (See Methods)	https://esgf-node.llnl.gov/search/input4mips/, last access 3/2/2024

Table S7. Model input data description and availability.

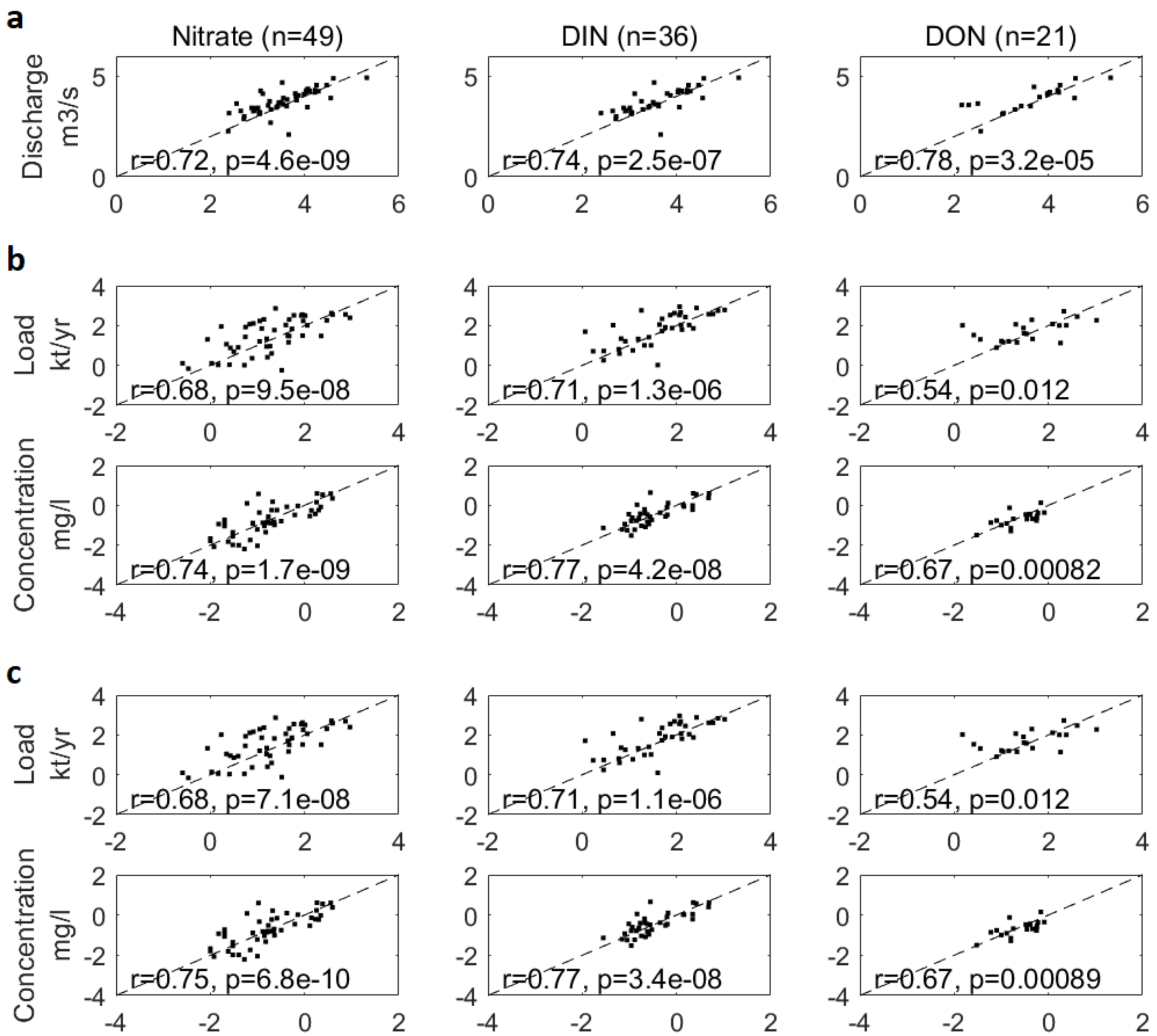

Fig. S1. **Model performance analysis of river water discharge and N. a-c**, Pearson correlation coefficients (r) and p values (p) between the log-transformed measurement-based vs. simulated river water discharge (**a**), N loads and concentrations without manure applications (**b**) and with manure applications (**c**) for nitrate, DIN, and DON across 49, 36, and 21 rivers for the year 1990 (Tables S1-S4).

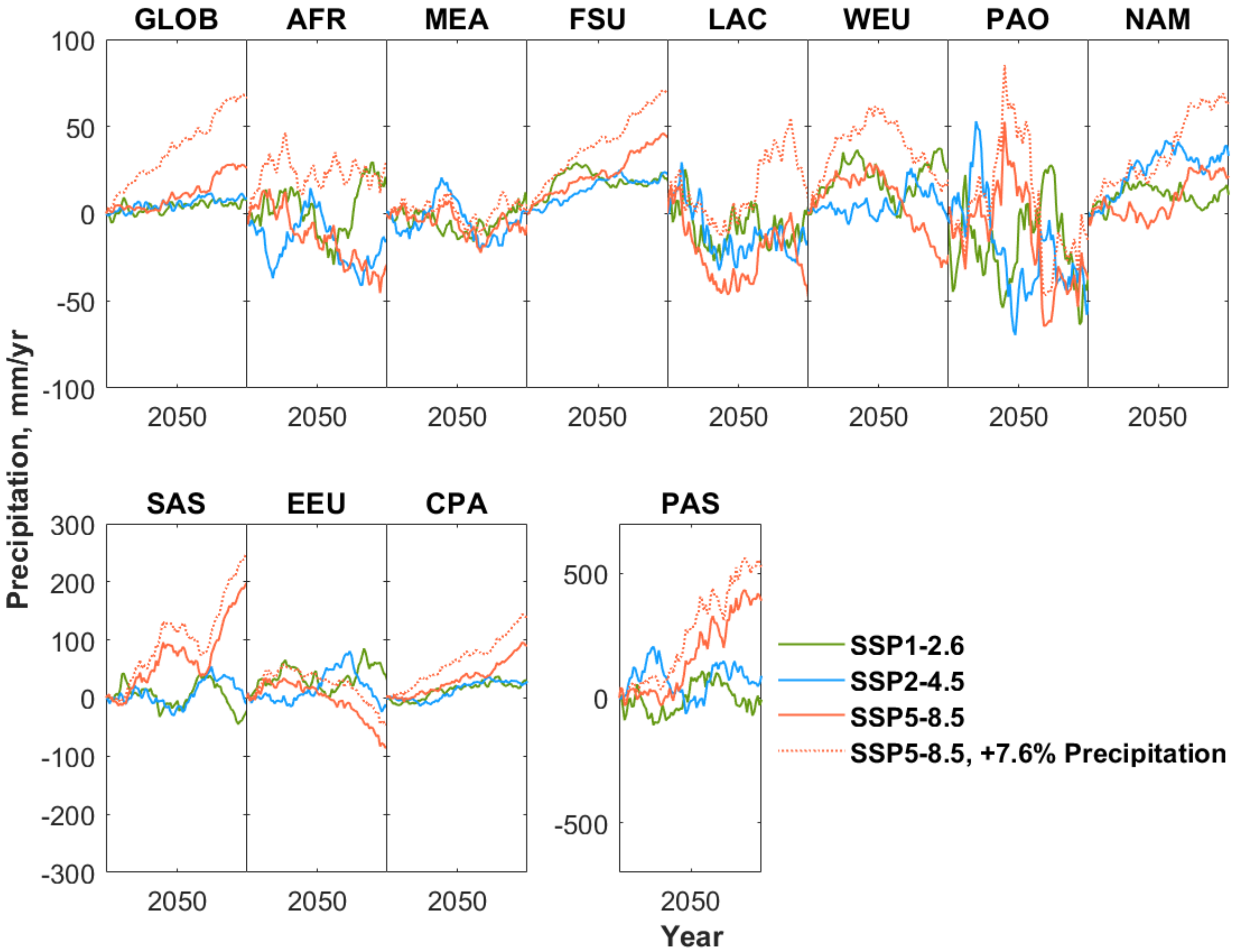

Fig. S2. Global and regional spatial mean precipitation for each of the marker scenarios and for the extreme precipitation uncertainty experiment. For the extreme precipitation experiment, precipitation in SSP5-8.5 was proportionally increased to yield a 7.6% increase by 2080-2099 relative to 1986-2005 (see Methods). This corresponds to the upper limit of projected precipitation changes under SSP5-8.5[31]. All plots show 21-year moving averages from 2000 to 2099 for global results and 11 aggregate regions: Sub-Saharan Africa (AFR), South Asia (SAS), Middle East and North Africa (MEA), Former Soviet Union (FSU), Central and Eastern Europe (EEU), Latin America and the Caribbean (LAC), Other Pacific Asia (PAS), Centrally Planned Asia and China (CPA), Western Europe (WEU), Pacific OECD (PAO), and North America (NAM). Source data are provided as a Source Data file.

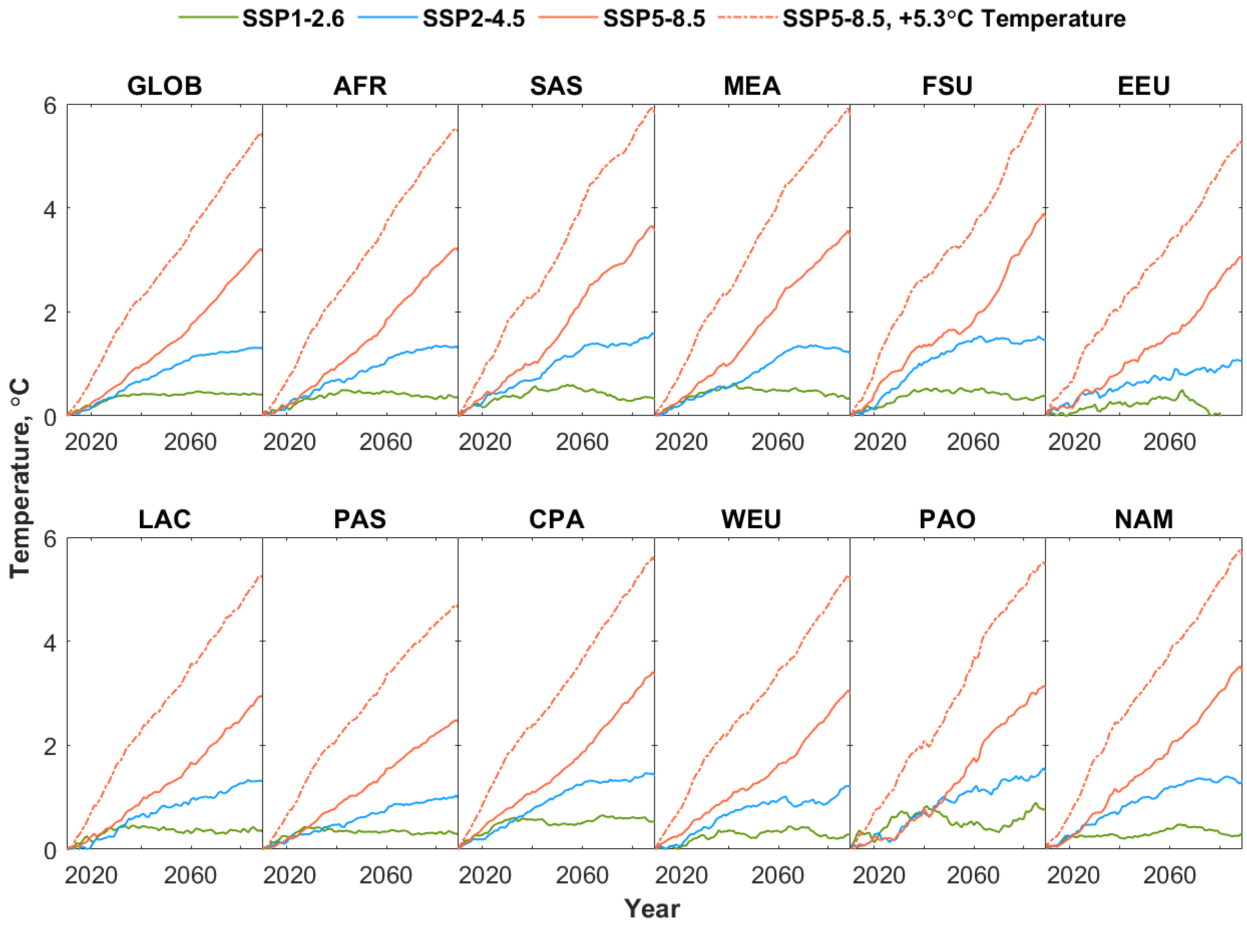

Fig. S3. Global and regional spatial mean temperature for each of the marker scenarios and for the extreme warming uncertainty experiment. In the extreme warming experiment, air temperature in SSP5-8.5 was proportionally increased to yield a 5.3°C increase by 2080-2099 relative to 1986-2005 (see Methods). This corresponds to the upper limit of projected temperature changes under SSP5-8.5[32]. All plots show 21-year moving averages from 2000 to 2099 for global results and 11 aggregate regions: Sub-Saharan Africa (AFR), South Asia (SAS), Middle East and North Africa (MEA), Former Soviet Union (FSU), Central and Eastern Europe (EEU), Latin America and the Caribbean (LAC), Other Pacific Asia (PAS), Centrally Planned Asia and China (CPA), Western Europe (WEU), Pacific OECD (PAO), and North America (NAM). Source data are provided as a Source Data file.

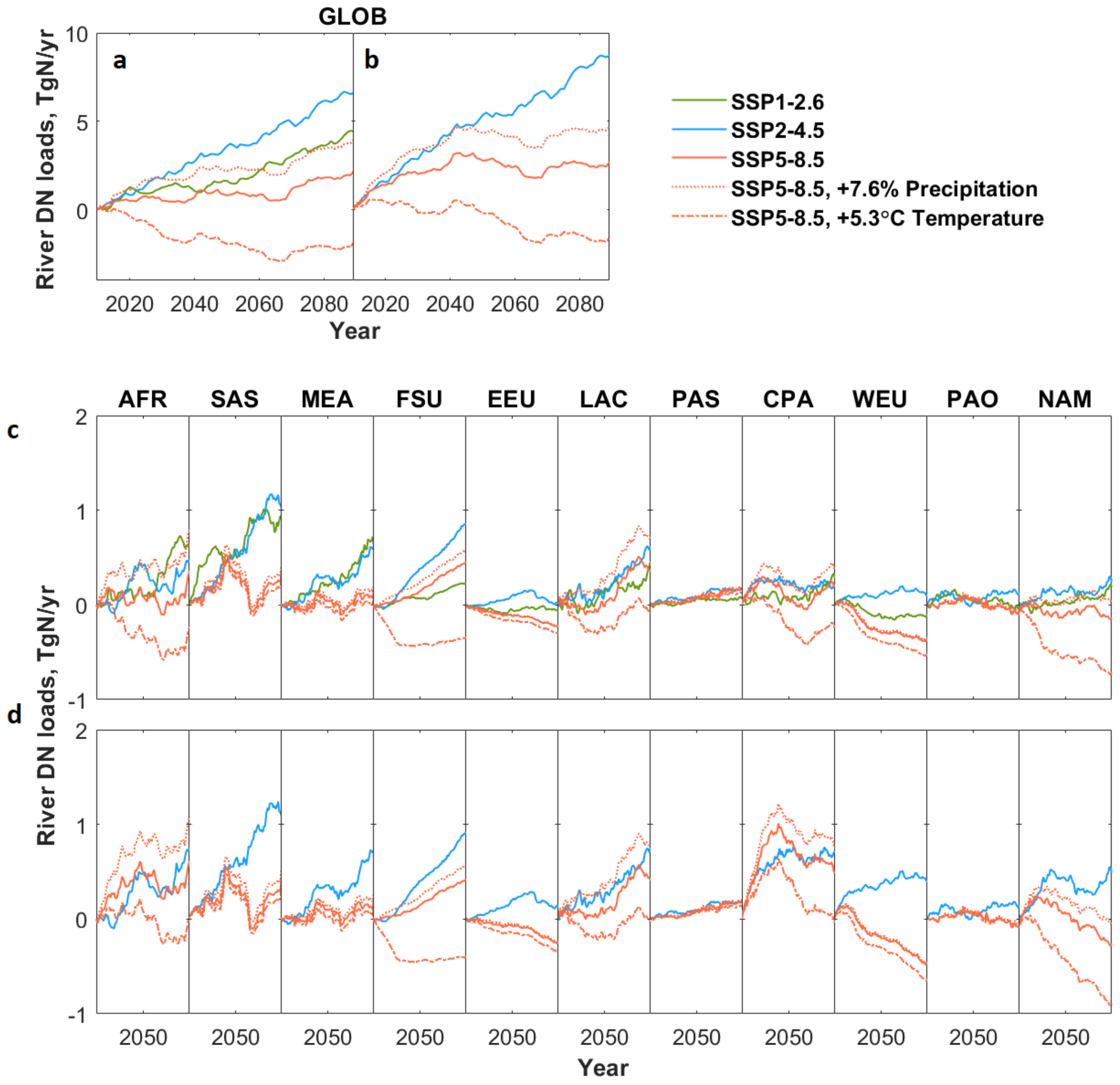

Fig. S4. Comparison of global and regional river DN loads for each of the marker scenarios and extreme climate change cases under SSP5-8.5. a-d, All plots show 21-year moving averages of river DN loads without manure applications (**a, c**) and with manure applications (**b, d**) from 2000 to 2099 for global results and 11 aggregate regions: Sub-Saharan Africa (AFR), South Asia (SAS), Middle East and North Africa (MEA), Former Soviet Union (FSU), Central and Eastern Europe (EEU), Latin America and the Caribbean (LAC), Other Pacific Asia (PAS), Centrally Planned Asia and China (CPA), Western Europe (WEU), Pacific OECD (PAO), and North America (NAM). Source data are provided as a Source Data file.

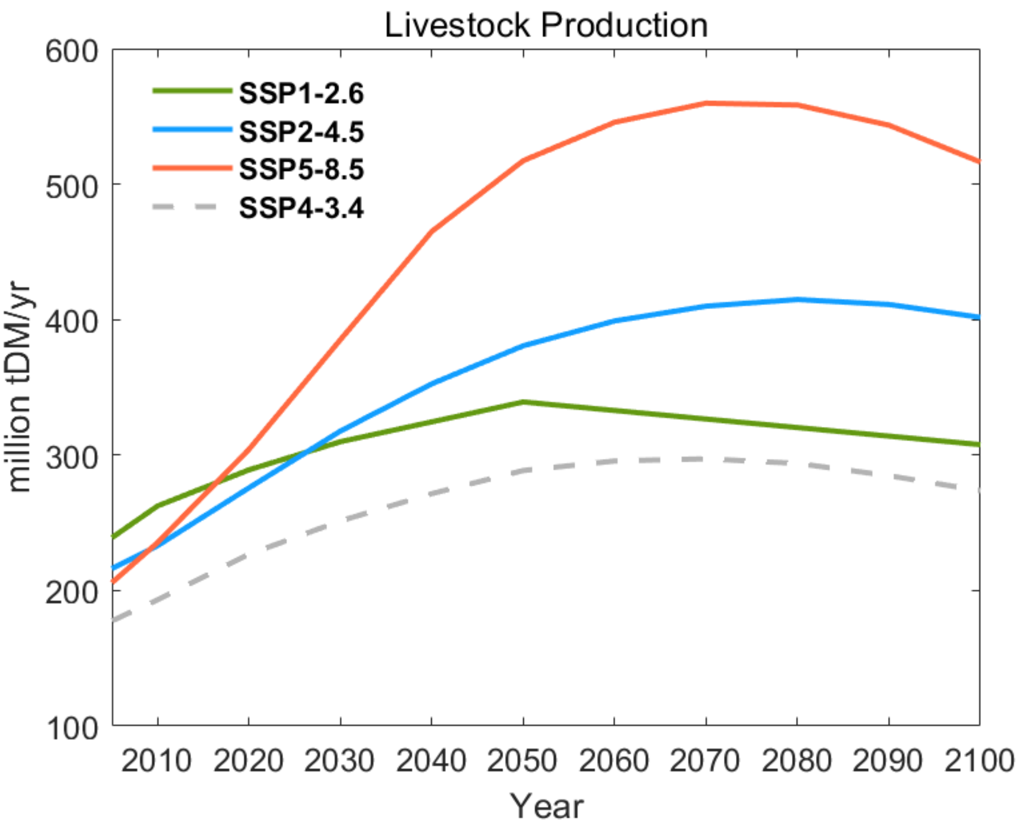

Fig. S5. Livestock production. These four IAM marker scenario projections are provided from the SSP Database[25] for the period 2005-2100. Source data are provided as a Source Data file.

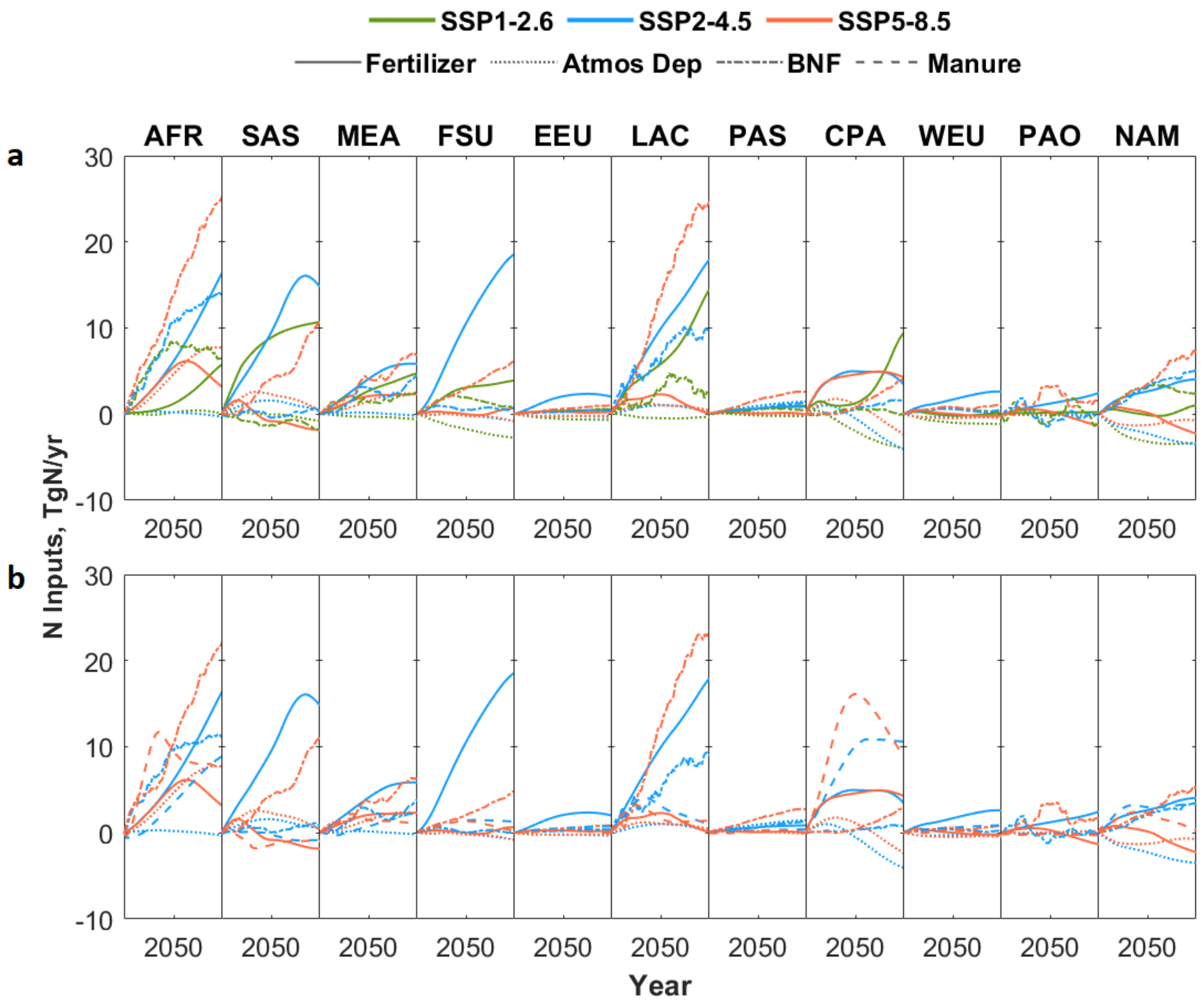

Fig. S6. N inputs. Projected fertilizer applications for each SSP depend on crop demands and agricultural productivity that impact the amount of fertilizer required per unit of land area[24,33]. Atmospheric deposition reflects a combination of energy demands, air pollutant controls, shares of renewables, energy efficiency improvements[34]. More detailed descriptions of the assumptions underlying each scenario can be found in references[27,29,30]. All plots show 21-year moving averages of inputs without manure applications (**a**) and with manure applications (**b**) from 2000 to 2099 for 11 aggregate regions: Sub-Saharan Africa (AFR), South Asia (SAS), Middle East and North Africa (MEA), Former Soviet Union (FSU), Central and Eastern Europe (EEU), Latin America and the Caribbean (LAC), Other Pacific Asia (PAS), Centrally Planned Asia and China (CPA), Western Europe (WEU), Pacific OECD (PAO), and North America (NAM). Source data are provided as a Source Data file.

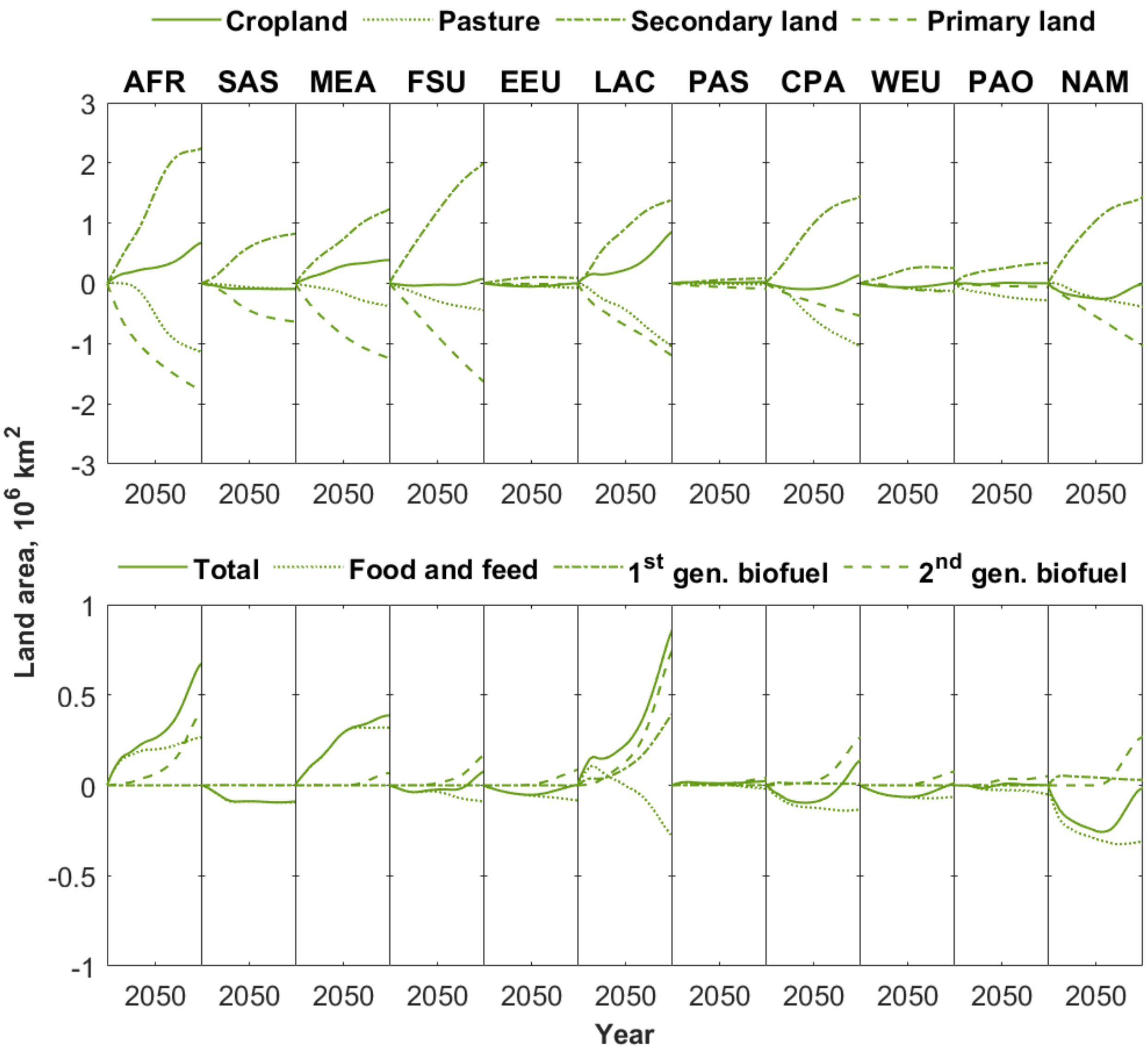

Fig. S7. Regional land area trajectories in SSP1-2.6. Primary lands are lands undisturbed by human activities during land-use reconstruction. Secondary lands are abandoned agricultural land or regrowing forest after logging (see Methods). Note that cropland area in SAS decreases while fertilizer usage increases substantially (Fig. S6). This indicates agricultural intensification to meet growing food demands. Such an increase in fertilize usage can be tempered by agricultural advances, such as agricultural yield increasing technologies (e.g., breeding, biotechnology traits), advances in agronomic practices (e.g., 4Rs: applying the right source of nutrients, at the right rate, at the right time, in the right place), and efficient livestock nutrition and waste management (e.g., shifts towards mixed crop-livestock systems)[35-37]. All plots show 21-year moving averages from 2000 to 2099 for 11 aggregate regions: Sub-Saharan Africa (AFR), South Asia (SAS), Middle East and North Africa (MEA), Former Soviet Union (FSU), Central and Eastern Europe (EEU), Latin America and the Caribbean (LAC), Other Pacific Asia (PAS), Centrally Planned Asia and China (CPA), Western Europe (WEU), Pacific OECD (PAO), and North America (NAM). Source data are provided as a Source Data file.

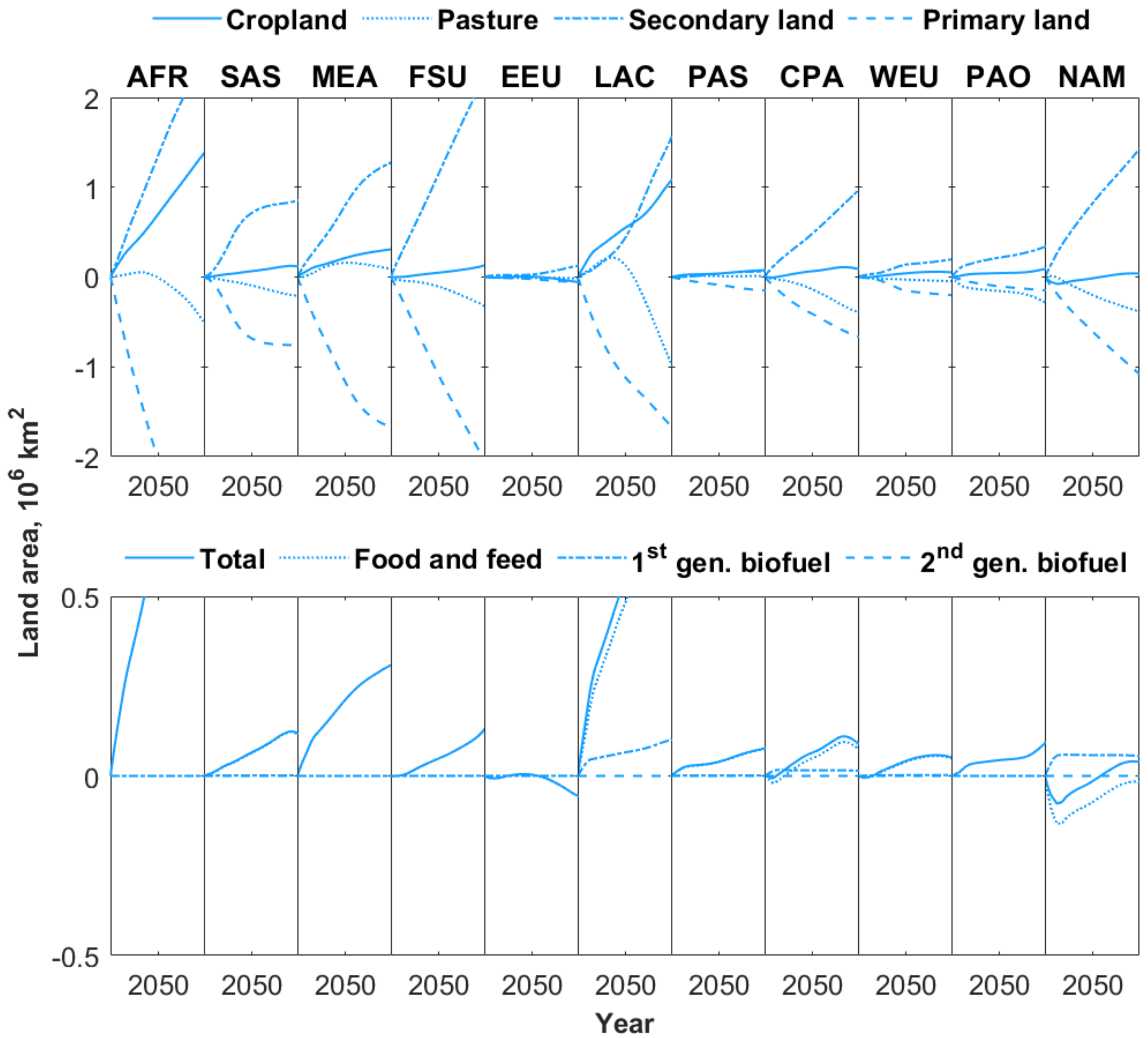

Fig. S8. Regional different cropland area trajectories in SSP2-4.5. We note that 2nd generation biofuel cropland areas in the LUH2 dataset[33] were reported as zeros, contrary to the documentation in Popp et al. (2017)[24] which identifies substantial increases in 2nd generation biofuel crop production in Africa, Asia and Latin America (see Fig. SI9 of Popp et al. (2017)[24]). Note that the extent of increases in fertilizer usage (Fig. S6) are often higher than those in cropland area. This can be explained by lower agricultural advances under SSP2-4.5 than SSP1-2.6 and SSP5-8.5[24]. All plots show 21-year moving averages from 2000 to 2099 for 11 aggregate regions: Sub-Saharan Africa (AFR), South Asia (SAS), Middle East and North Africa (MEA), Former Soviet Union (FSU), Central and Eastern Europe (EEU), Latin America and the Caribbean (LAC), Other Pacific Asia (PAS), Centrally Planned Asia and China (CPA), Western Europe (WEU), Pacific OECD (PAO), and North America (NAM). Source data are provided as a Source Data file.

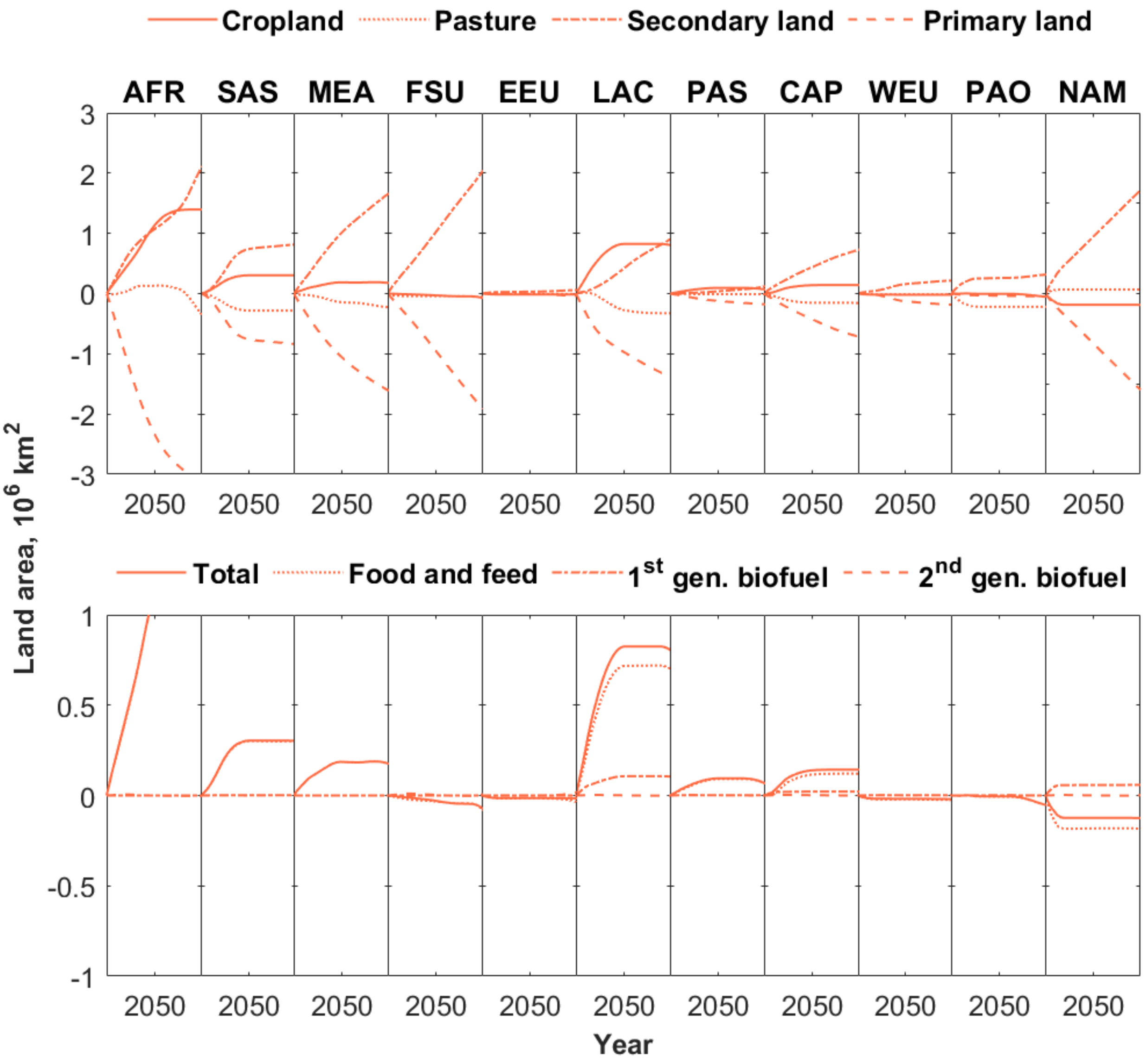

Fig. S9. Regional different cropland area trajectories in SSP5-8.5. All plots show 21-year moving averages from 2000 to 2099 for 11 aggregate regions: Sub-Saharan Africa (AFR), South Asia (SAS), Middle East and North Africa (MEA), Former Soviet Union (FSU), Central and Eastern Europe (EEU), Latin America and the Caribbean (LAC), Other Pacific Asia (PAS), Centrally Planned Asia and China (CPA), Western Europe (WEU), Pacific OECD (PAO), and North America (NAM). Source data are provided as a Source Data file.

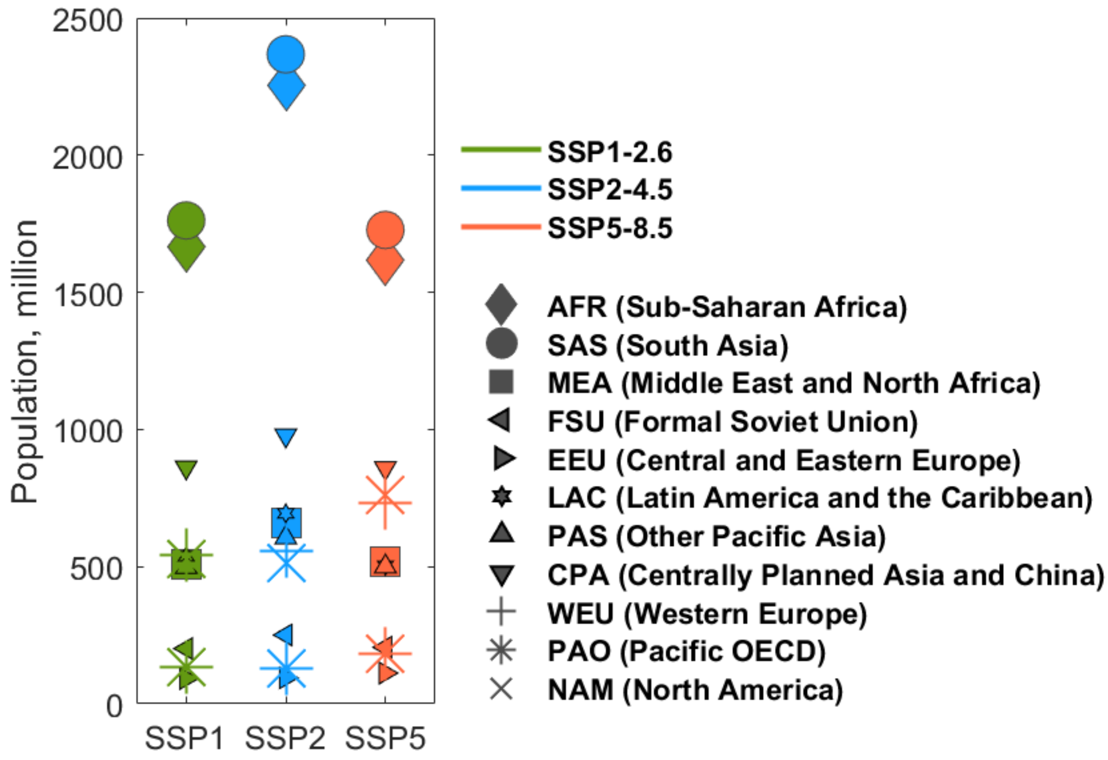

Fig. S10. Regional population. All plots show 2080-2100 average population[25,38]. Source data are provided as a Source Data file.

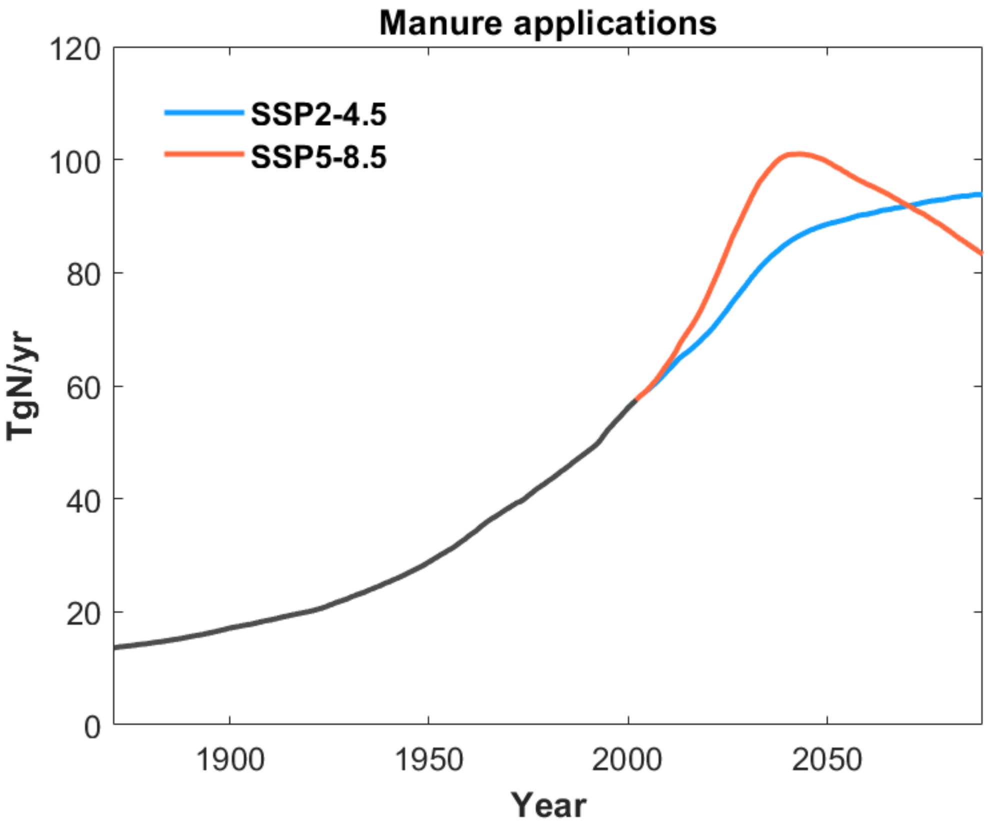

Fig. S11. Manure applications. Simulated manure applications for the period 1861-2001 (Note S2) and reported manure applications for the period 2002-2099[19-21] in 21-year moving averages. Source data are provided as a Source Data file.

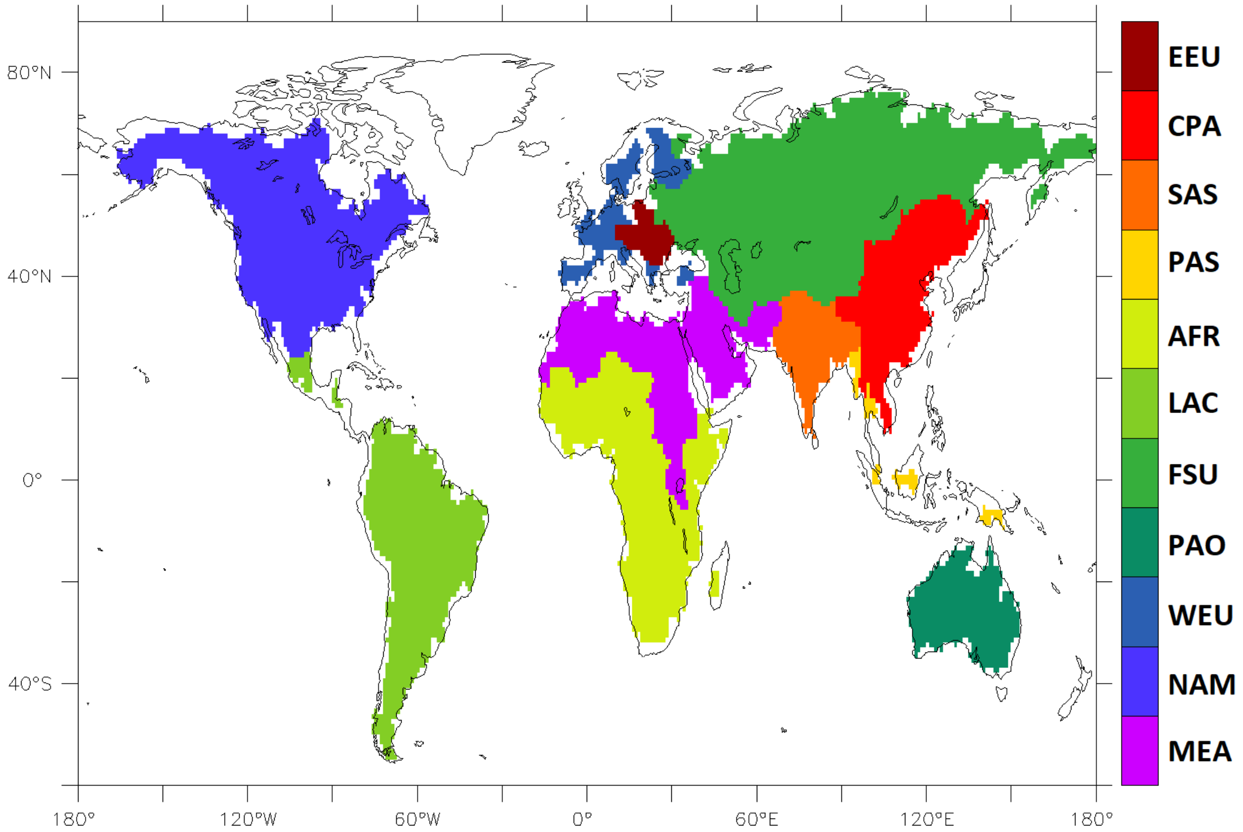

Fig. S12. **11 aggregate regions.** The results are presented and discussed for 11 aggregate regions: Sub-Saharan Africa (AFR), South Asia (SAS), Middle East and North Africa (MEA), Former Soviet Union (FSU), Central and Eastern Europe (EEU), Latin America and the Caribbean (LAC), Other Pacific Asia (PAS), Centrally Planned Asia and China (CPA), Western Europe (WEU), Pacific OECD (PAO), and North America (NAM). Source data are provided as a Source Data file.

References

1. Harrison, J. A., Caraco, N. & Seitzinger, S. P. Global patterns and sources of dissolved organic matter export to the coastal zone: Results from a spatially explicit, global model. *Global Biogeochem. Cy.* **19**, GB4S04 (2005).
2. Meybeck, M. & Ragu, A. GEMS-GLORI world river discharge database, https://doi.org/10.1594/PANGAEA.804574, 1085 (2012).
3. Lee, M., Shevliakova, E., Stock, C. A., Malyshev, S. & Milly, P. C. D. Prominence of the tropics in the recent rise of global nitrogen pollution. *Nat. Commun.* **10**, 1437 (2019).
4. Lee, M., Malyshev, S., Shevliakova, E., Milly, P. C. D. & Jaffé, P. R. Capturing interactions between nitrogen and hydrological cycles under historical climate and land use: Susquehanna Watershed analysis with the GFDL Land Model LM3-TAN. *Biogeosciences* **11**, 5809–5826 (2014).
5. Bowie, G. L., Mills, W. B., Porcella, D. B., Campbell, C. L., Pagenkopf, J. R., Rupp, G. L., Johnson, K. M., Chan, P. W. H. & Gherini, S. A. Rates, constants, and kinetics formulations in surface water quality modeling (Athens, Georgia, 1985).
6. Alexander, R. B. et al. Dynamic modeling of nitrogen losses in river networks unravels the coupled effects of hydrological and biogeochemical processes. *Biogeochem.* **93**, 91–116 (2009).
7. Randall, D. A. et al. *In Climate Change 2007: The Physical Science Basis* (eds Solomon, S. D. et al.) Cilmate Models and Their Evaluation (Cambridge University Press, Cambridge, United Kingdom and New York, NY, USA, 2007).
8. Mayorga, E., Seitzinger, S. P., Harrison, J. A., Dumont, E., Beusen, A. H. W., Bouwman, A. F. Fekete, B. M., Kroeze, C. & Van Drecht, G. Global Nutrient Export from WaterSheds 2 (NEWS 2): Model development and implementation. *Environ. Model. Softw.* **25**, 837-853 (2010).
9. Green, P. A., Vörösmarty, C. J., Meybeck, M., Galloway, J. N., Peterson, B. J. & Boyer, E. W. Pre-industrial and contemporary fluxes of nitrogen through rivers: a global assessment based on typology. *Biogeochemistry* **68**, 71–105 (2004).
10. Post, W. M., Pastor, J., Zinke, P. J. & Stangenberger, A. G. Global patterns of soil nitrogen storage. *Nature* **317**, 613–616 (1985).
11. Smith, S. V., Swaney, D. P., Talaue-Mcmanus, L., Bartley, J. D., Sandhei, P. T., McLaughlin, C. J., Dupra, V. C., Crossland, C. J., Buddemeier, R. W., Maxwell, B. A. & Wulff, F. Humans, Hydrology, and the Distribution of Inorganic Nutrient Loading to the Ocean. *BioScience* **53**, 234-245 (2003).
12. Ciais, P. C. et al. in Climate Change 2013: The Physical Science Basis (eds Stocker, T. F. et al.) 465–570 (Cambridge Univ. Press, Cambridge, 2013).
13. Khatiwala, S., Primeau, F. & Hall, T. Reconstruction of the history of anthropogenic CO2 concentrations in the ocean. *Nature* **462**, 346–350 (2009).
14. Peylin, P. et al. Global atmospheric carbon budget: results from an ensemble of atmospheric CO2 inversions. *Biogeosciences* **10**, 6699–6720 (2013).
15. Schimel, D., Stephens, B. B. & Fishera, J. B. Effect of increasing CO2 on the terrestrial carbon cycle. *Proc. Natl Acad. Sci. USA* **112**, 436–441 (2015).
16. Lee, M., Shevliakova, E., Malyshev, S., Milly, P. C. D., & Jaffé, P. R. Climate variability and extremes, interacting with nitrogen storage, amplify eutrophication risk. Geophys. Res. Lett. 43, https://doi.org/10.1002/2016GL069254 (2016).

17. Lee, M., Stock, C. A., Shevliakova, E., Malyshev, S. & Milly, P. C. D. Globally prevalent land nitrogen memory amplifies water pollution following drought years. *Environ. Res. Lett.* **16** 014049 (2021).
18. Lee, M. et al. Control of nitrogen exports from river basins to the coastal ocean: Evaluation of basin management strategies for reducing coastal hypoxia. *J. Geophys. Res. Biogeosci.* **123**, 3111–3123 (2018).
19. Beaudor, M. et al. Global agricultural ammonia emissions simulated with the ORCHIDEE land surface model. *Geosci. Model Dev.* **16**, 1053–1081 (2023a).
20. Beaudor, M., Vuichard, N., Lathière, J. & Hauglustaine, D. Global ammonia emissions from CAMEO throughout the century for 3 scenarios (2000-2100), https://zenodo.org/records/10100435 (2023b).
21. Beaudor, M. Vuichard, N., Lathière, J. & Hauglustaine, D. Future trends of agricultural ammonia global emissions in a changing climate. *ESS Open Archive*, https://doi.org/10.22541/essoar.170542263.35872590/v1 (2024).
22. Meybeck, M., & Ragu, A. River Discharges to the Oceans: An Assessment of Suspended Solids, Major Ions, and Nutrients Environment Information and Assessment Technical Report (U.N. Environ. Programme, Nairobi 1996).
23. Barron, A. R. Patterns and controls of nitrogen fixation in a lowland tropical forest, Panama. Ph.D. diss., Princeton University, Princeton, NJ, USA (2007).
24. Popp, A. et al. Land-use futures in the shared socio-economic pathways. *Global Environ. Change* **42**, 331–345 (2017).
25. SSP Database. https://tntcat.iiasa.ac.at/SspDb/dsd?Action=htmlpage&page=10 (2023).
26. Calvin, K. et al. The SSP4: A world of deepening inequality. *Glob. Environ. Change* **42**, 284-296 (2017).
27. Fricko, O. et al. The marker quantification of the Shared Socioeconomic Pathway 2: A middle-of-the-road scenario for the 21st century. *Global Environ. Change* **42**, 251–267 (2017).
28. Fujimori, S. et al. SSP3: AIM implementation of Shared Socioeconomic Pathways, *Global Environ. Change* **42**, 268-283 (2017).
29. Kriegler, E. et al. Fossil-fueled development (SSP5): an energy and resource intensive scenario for the 21st century. *Global Environ. Change* **42**, 297–315 (2017).
30. van Vuuren, D. P. et al. Energy, land-use and greenhouse gas emissions trajectories under a green growth paradigm. *Global Environ. Change* **42**, 237–250 (2017).
31. Intergovernmental Panel on Climate Change (IPCC) *Climate Change 2007: The Physical Science Basis* (eds Solomon, S. D. et al.) https://www.ipcc.ch/site/assets/uploads/2018/05/ar4_wg1_full_report-1.pdf (Cambridge University Press, Cambridge, United Kingdom and New York, NY, USA, 2007).
32. Intergovernmental Panel on Climate Change (IPCC) *Climate Change 2014: Impacts, Adaptation, and Vulnerability* (eds Field, C. B.) (Cambridge University Press, Cambridge, United Kingdom and New York, NY, USA, 2014).
33. Hurtt, G. C. et al. Harmonization of global land use change and management for the period 850–2100 (LUH2) for CMIP6 Geoscientific Model Development. *Geosci. Model Dev.* **13**, 5425–5464 (2020).
34. Rao, S. et al. Future air pollution in the Shared Socio-economic Pathways. *Global Environ. Change* **42**, 346–358 (2017).

35. Dietrich, J. P., Schmitz, C., Lotze-Campen, H., Popp, A. & Müller, C. Forecasting technological change in agriculture—An endogenous implementation in a global land use model. *Technol. Forecast Soc. Change* **81**, 236-249 (2014).
36. Edgerton, M. D. Increasing Crop Productivity to Meet Global Needs for Feed, Food, and Fuel. *Plant Physiol.* **149**, 7–13 (2009).
37. Weindl, I. et al. Livestock in a changing climate: production system transitions as an adaptation strategy for agriculture. *Environ. Res. Lett.* **10**, 094021 (2015).
38. KC, S. & Lutz, W. The human core of the shared socioeconomic pathways: Population scenarios by age, sex and level of education for all countries to 2100. *Global Environ. Change* **42**, 181–192 (2017).
